# American Academy of Dental Science

**Published:** 1893-03

**Authors:** 

**Affiliations:** American Academy of Dental Science


### AMERICAN ACADEMY OF DENTAL SCIENCE.

The regular monthly meeting of the American Academy of Dental Science was held in the Boston Medical Library Association rooms on December 7, 1892, at 7.30 P.M., President Brackett in the chair.

The paper of the evening was read by Dr. James A. Reilly; subject, "Unnecessary Pain in Dental Operations."

(For Dr. Reilly's paper, see page 164.)

DISCUSSION.

*Dr. Brackett.*—I heard a remark some years ago concerning a dental practitioner, that all his patients became his friends. If the remark were true, as I suppose it was, the dentist did not attain that end by disregard of such advice as Dr. Reilly has put before us. If there is no discussion of this paper, the next thing in order is the exhibition of models of a case of regulating with the appliances that were used by Dr. Smith.

*Dr. Smith.*—This case was treated in the Infirmary of the Harvard Dental School. The lateral incisors were crowded far inside the arch by the cuspids, and there might be a difference of opinion as to what teeth should be extracted. In this case I advised the student having charge of the operation to extract the first upper bicuspids. After that was done, an appliance was made to move the canines back into the places occupied by the first bicuspids. This appliance consisted of two Magill bands soldered together, and cemented to the bicuspid and molar on either side. On the buccal side of these bands was attached a screw, while the palatal surface held a small hook. To this hook a strong ligature was fastened and passed around a Magill band, which had been cemented to the cuspids and thence to the screw. By turning the screw backward the ligature was tightened, and pressure brought to bear upon the cuspids, which were slowly brought to place. The next step was to cement Magill bands to the laterals. To these bands were attached screws and nuts, the screws extending outward through an opening in a bar, which reached from cuspid to central. This appliance brought the laterals into line.

*Dr. Williams.*—I simply want to say that I am very glad to see that one principle has become generally recognized,—namely, that rest is as important as motion in cases of regulation.

*President Brackett.*—I understand Dr. Smith that the ligature used was silk. There was no elastic or rubber in any part of the appliance?

*Dr. Smith.*—No elastic or rubber. The screw is moved very gradually. I always caution students not to go too fast. You cannot move a tooth as you would a building on the street. With so much power one must be careful. I had a student bring out a lateral incisor from inside the arch in four days. It simply shows what a student will do if left alone with such an appliance. You can move a tooth more rapidly than you can produce absorption. Teeth should not be moved any faster than absorption takes place, unless the intention be to take advantage of the elasticity of the alveolar and spring it.

*Dr. Fillebrown.*—Here is the model of a case of bridge-work done some years ago. There are no Richmond crowns in connection with it. The work has proved both useful and durable to the wearer. The patient was about seventy years of age, a man of very nervous temperament, and his teeth were worn short and thin. He had lost an upper first and second molar on both sides, and wished to have something done to make his teeth more useful. The lost molars had to be supplied, and the bicuspids, being in poor condition, needed crowns. It was out of the question to grind the third molars for the fitting of caps around the margin of the gum. A way occurred to me, therefore, of making a cap and putting it on without grinding down the teeth. I first passed a band of pure gold, about number thirty, around the tooth, and towards the grinding surface added a second thickness of coin-gold. The pure gold was burnished to the tooth when the case was cemented on. The case has proved a very desirable and useful thing for my patient. My confidence in bridge-work is strong, and has grown steadily from the first. I am glad to say that the first case I did, about 1880, has shown good service. It has never been taken off. I know of but few instances where I have had to make many repairs. I have had some failures, from decay of roots of teeth or from absorption of socket. In one or two cases the bridge-work has failed. Less than a half-dozen cases have made me any trouble.

WILLIAM H. POTTER, D.M.D.,
*Editor American Academy of Dental Science.*

